# Safety, Immunogenicity and Duration of Immunity Elicited by an Inactivated Bovine Ephemeral Fever Vaccine

**DOI:** 10.1371/journal.pone.0082217

**Published:** 2013-12-12

**Authors:** Orly Aziz-Boaron, Keren Leibovitz, Boris Gelman, Maor Kedmi, Eyal Klement

**Affiliations:** 1 Koret School of Veterinary Medicine, The Robert H. Smith Faculty of Agriculture, Food and Environment, The Hebrew University, Jerusalem, Israel; 2 Kimron Veterinary Institute, Beit-Dagan, Israel; 3 Hachaklait Veterinary Services Ltd., Ceasarea, Israel; Thomas Jefferson University, United States of America

## Abstract

Bovine ephemeral fever (BEF) is an economically important viral vector-borne cattle disease. Several live-attenuated, inactivated and recombinant vaccines have been tested, demonstrating varying efficacy. However, to the best of our knowledge, duration of immunity conferred by an inactivated vaccine has never been reported. In the last decade, Israel has faced an increasing number of BEF outbreaks. The need for an effective vaccine compatible with strains circulating in the Middle East region led to the development of a MONTANIDE™ ISA 206 VG (water-in-oil-in-water), inactivated vaccine based on a local strain. We tested the safety, immunogenicity and duration of immunity conferred by this vaccine. The induced neutralizing antibody (NA) response was followed for 493 days in 40 cows vaccinated by different protocols. The vaccine did not cause adverse reactions or a decrease in milk production. All cows [except 2 (6.7%) which did not respond to vaccination] showed a significant rise in NA titer of up to 1:256 following the second, third or fourth booster vaccination. Neutralizing antibody levels declined gradually to 1:16 up to 120 days post vaccination. This decline continued in cows vaccinated only twice, whereas cows vaccinated 3 or 4 times showed stable titers of approximately 1:16 for up to 267 days post vaccination. At least three vaccinations with the inactivated BEF vaccine were needed to confer long-lasting immunity. These results may have significant implications for the choice of vaccination protocol with inactivated BEF vaccines. Complementary challenge data should however be added to the above results in order to determine what is the minimal NA response conferring protection from clinical disease.

## Introduction

Bovine ephemeral fever (BEF) is an economically important disease in cattle and buffalo, characterized by biphasic fever, anorexia, lameness and recumbency [1]. The disease is caused by a vector-borne single-stranded RNA virus—bovine ephemeral fever virus (BEFV)—and inflicts significant economic losses, mainly due to reduction in milk production [2]. 

 Since the exact vector of BEF has not been identified, prevention efforts are mainly aimed at efficient vaccination of susceptible animals. The earliest BEF vaccines were based on field isolates of BEFV which were attenuated by repeated passages in suckling mice and/or cell cultures [3]. These vaccines were prepared with various adjuvants such as Freund’s complete or incomplete adjuvant, aluminum hydroxide, dextran sulfate, or Quil A [4-6]. Many of the live attenuated (LA) vaccines produced a long-lasting neutralizing antibody (NA) response which lasted more than 12 months after two vaccinations. These vaccines demonstrated variable protection from clinical disease after both experimental [4,7] and natural challenge [6]. 

Though commercial LA vaccines have been used in many endemic countries [4,5,7,8], their use is discouraged by some due to their potential lack of safety. The fact that these vaccines contain attenuated live viruses carries the risk that these viruses might back-mutate to their virulent form [9], especially considering the relatively high mutation rate of RNA viruses [10]. Furthermore, as these vaccines are not inactivated, and their preparation involves the use of materials of biological origin, there is also the potential for contamination with other viruses [11,12]. Therefore, the use of LA vaccines produced in one geographical region requires a careful risk assessment prior to their introduction into new regions [13]. Other weaknesses of LA vaccines include their potential for causing adverse clinical reactions [14] and their potential sensitivity to impairment by heat or light. Thus, an important practical concern has been raised regarding the use of these vaccines in countries where maintenance can be extremely difficult.

 The use of inactivated vaccines is considered a safer approach. In the process of inactivation, the pathogen’s ability to propagate in the vaccinated host is destroyed but the viral capsid remains intact, such that it is still recognized by the immune system. Inactivation of BEFV has been achieved using a variety of agents such as formalin [8], β-propiolactone [15], and binary ethyleneimine [16]. Several adjuvants have been used for inactivated BEFV vaccines. These include aluminum phosphate gel, Freund's incomplete adjuvant and water-in-oil-in-water (w/o/w) emulsions. Though these vaccines provide variable protection against challenge, the NA levels they induce have been shown to wane rapidly after the first vaccination [8,15]. An exception was observed with the Quil A adjuvanted vaccine, which provided protection 12 months after vaccination and the induction of a high NA response after both experimental challenge and natural exposure in the field. However, this vaccine cannot be regarded as fully inactivated, as inactivation by Quil A is not complete [6,17]. 

 Attempts to develop other vaccines have also been made. A subunit vaccine based on G protein was developed [18] and was found to provide protection from disease but not from infection. The virus-vector vaccine is the most recently developed approach, using nonpathogenic live virus as a delivery vehicle for foreign DNA, inducing a sufficient immunity response against the inserted proteins. Such a recombinant vaccine was constructed based on the insertion of BEFV G protein into the South African vaccine strain of lumpy skin disease virus [19]. In a small-scale BEFV-challenge cattle trial, this construct failed to protect against virulent challenge. Today, the virus-vector and subunit-based vaccines are not used commercially and further research is needed to explore their potential. 

 In the last decade, Israel has been facing a dramatic increase in the number of BEF outbreaks [20-22]. As indicated by phylogenetic analysis, significant differences have been found between the field strains circulating in the Middle East and other isolates [23]. The need for a vaccine which is antigenically similar to the field strains circulating in the Middle East led to the development of an experimental inactivated vaccine based on an Israeli strain mixed with the adjuvant MONTANIDE™ ISA 206 VG (w/o/w). In this study, we describe the safety of this vaccine and the dynamics of NA response following vaccination by several protocols.

## Materials and Methods

### 1: Ethical statement

 The study took place in a dairy herd of high-producing Israeli Holstein cows located in the southern Coastal Plain region in Israel after permission from the owner and was conducted with his cooperation. This study was carried out in strict accordance with the recommendations in the Guide for the Care and Use of Laboratory Animals of the National Institutes of Health. The study protocol was approved by the Institutional Animal Care and Use Committee of the Hebrew University of Jerusalem, Israel. (Approval number: MD-12-13280-2). All efforts were made to minimize animal suffering. Blood was drawn according to the accepted practice by venipuncture of the medial coccygeal vein, using 18 gauge needles and 10 ml containers (Vacutainer®, Becton, Dickinson and Company©). Restraint was applied by self-closing yokes abundant in the stable for the application of medical treatment and rectal examinations. 

### 2: Virus and cells

 The BEFV strain Yaqum-00 was used for vaccine preparation. This virus was isolated from an infected febrile cow on a farm located in the Israeli coastal plain during the 2000 outbreak. Virus was initially propagated using Vero cells (green monkey kidney cells- CLS order no. 605372). After five passages, the virus was adapted to the Baby Hamster Kidney cell line (BHK)-21 cell line (ATCC^®^ CCL-10^TM^). The cell line was cultured in Dulbecco modified Eagle medium (DMEM) supplemented with 10% (v/v) heat-inactivated fetal bovine serum (FBS) and 1% (v/v) of an antibiotic mix (penicillin, streptomycin, amphotericin B) and 10% (v/v) tryptose-phosphate broth. All cell lines were cultured in a humidified atmosphere containing 5% CO_2_ at 37°C. After the appearance of cytopathic effects, virus identification was reconfirmed using RT-PCR followed by sequence analysis. 

### 3: RT-PCR

Total RNA was extracted using the QIAamp Viral RNA Mini Kit (Qiagen, Valencia, CA, USA) to obtain a sufficient viral load for the subsequent analyses and replications. RNA was reverse-transcribed to cDNA using the Verso cDNA Kit (Thermo Fisher Scientific, Surrey, UK). PCR amplification of the conserved G gene was successfully performed on all isolates using GoTaq Green Master Mix (Promega, Madison, WI, USA) with two-step PCR using the primer set: 

 BEF346F 5'-TATTACCCTCCTGCCGGATGCTTT-3'

BEF1155R 5'-AGGTCTGTATTCGCACCAAGCTCT-3'.

Thermal cycling conditions for the first PCR were: denaturation at 95°C for 5 min, annealing at 56°C for 1 min and strand elongation at 72°C for 1 min followed by 30 to 35 cycles with denaturation at 95°C for 30 s. For final elongation, an additional step at 72°C for 10 min was applied.

### 4: BEFV vaccine preparation

BEFV vaccine was developed at the Kimron Veterinary Institute. The virus was inoculated onto a BHK-21 monolayer for 30–35 h and harvested at over 70% cytopathic effect. The tissue-culture infectious dose was assessed by titration to be in the range of 10^45^.–10^55^. TCID_50_/ml. Virus was filtered through a 5.0-µm filter and was inactivated for 30 h with 0.0125% β-propiolactone. Inactivation was stopped by the addition of 20% sodium thiosulfate. Inactivated antigen was then frozen (-70°C) and thawed, then centrifuged at 9,600*g* for 15 min. Antigen was then concentrated using a Hollow Fiber Cartridge of 30,000D followed by filtration of the supernatant using a 0.45-µm filter. The inactivated antigen was tested for sterility in the following media: thioglycollate, trypticase soy broth and agar blood plates. Antigen was mixed for 10 minutes using a stirrer with MONTANIDE^TM^ ISA 206 VG oil adjuvant (*SEPPIC*, FRANCE), heated to 37°C and filtered through a 0.45-µm filter. The vaccine was divided into flasks and kept at 4°C until use. 

### 5: Safety and immunogenicity study

**Figure 1:**
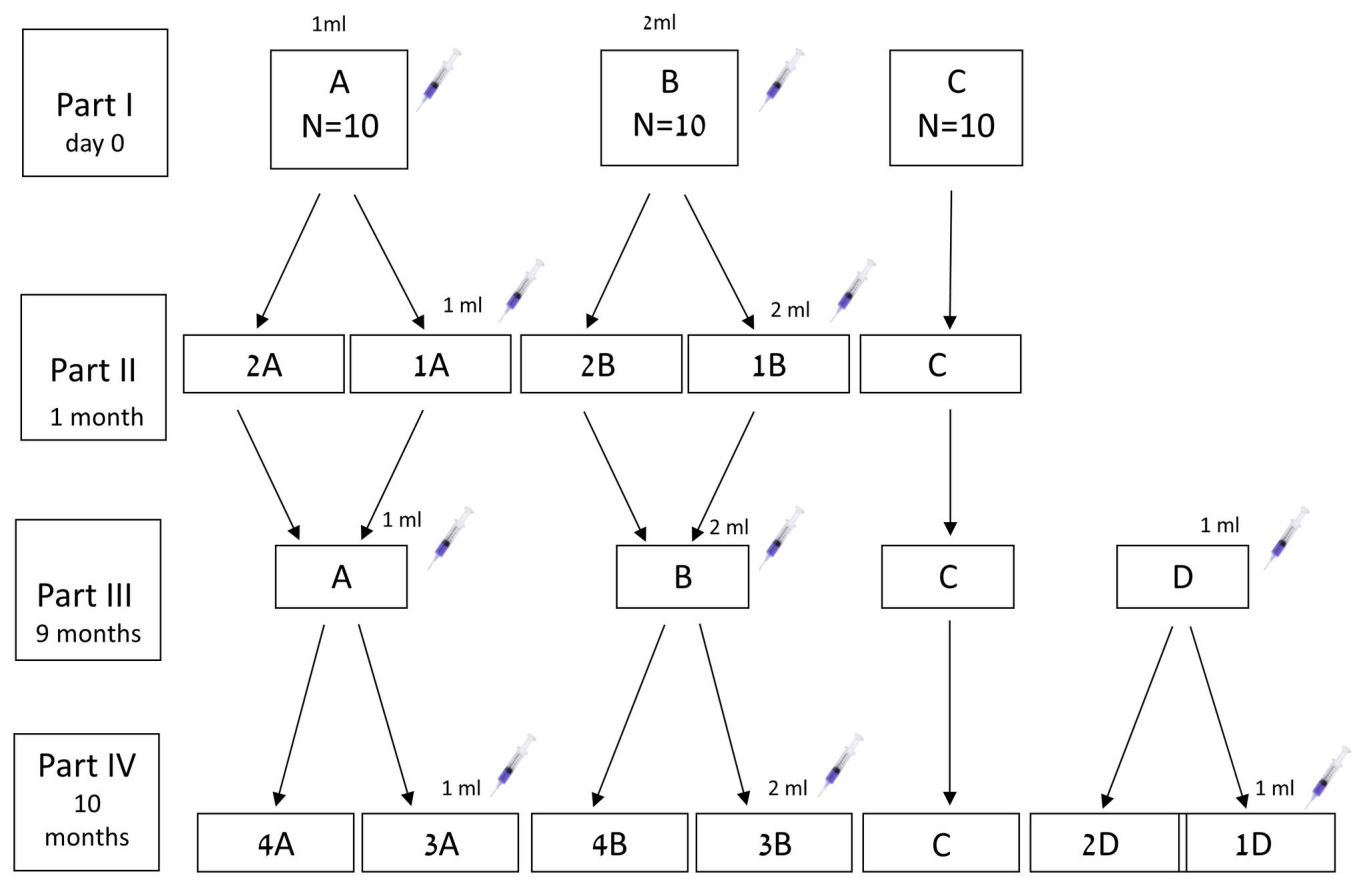
Study flow diagram. Part I: study initiation. intramuscular vaccination: Group A; 1-ml vaccine, Group B; 2 -ml vaccine and Group C; non vaccinated. Part II: 1 month (30 days) post-study initiation (PSI), a second vaccination dose was administered to half of each vaccinated group as follows: A1; 1-ml, B1; 2- ml. Part III: 9 months (267 days) PSI, Group A was injected with 1 ml vaccine, Group B with 2 ml vaccine, Group C was not vaccinated and another group (Group D) was vaccinated with 1- ml. Part IV: 10 months (295 days) PSI, another vaccination dose was administered to half of each vaccinated group as follows: A3; 1- ml, B3; 2- ml, D1; 1- ml.

The study flow diagram is depicted in Figure **1**. Three days before vaccine administration, all cows were thoroughly examined for any underlying morbidity. Only cows that were found to be perfectly healthy were included in the study. Thirty Holstein dairy cows free of anti-BEFV NAs were randomly divided into three groups: 10 cows were vaccinated intramuscularly with a 1-ml dose of the vaccine (Group A); 10 cows were vaccinated with a 2-ml dose of the vaccine (Group B) and 10 non vaccinated cows served as controls (Group C). One month later [30 days post-study initiation (PSI)], groups A and B were divided into two subgroups (A1 and A2, and B1 and B2, respectively). Groups A1 and B1 were vaccinated with the same initial vaccine dose. Nine months later (267 days PSI), all of the cows in groups A and B were vaccinated with the same respective vaccine dose and after another month (295 days PSI) half of each group was vaccinated again. On day 267 PSI, another group of 10 cows was vaccinated with a 1-ml dose of the vaccine (Group D). Half of this group (D1) was vaccinated again on day 295 PSI. 

All cows were subjected to normal herd management on the farm and vaccine safety was evaluated after first vaccination according to the following protocol: on the day of vaccination, all cows were clinically examined. Rectal temperature was examined daily from the day of vaccination until 7 days after vaccination and the injection area was inspected to detect local reactions. Milk production was monitored from 7 days prior to vaccination until 6 days after vaccination. 

 Blood samples were obtained from each cow in the study on the following days PSI: 0, 7, 14, 21, 30, 36, 43, 60, 169, 267, 274, 282, 295, 309, 325, 353, 419, 493. Sera were separated from whole blood and kept at -20°C until performance of the serum neutralization (SN) assay. 

### 6: Serum neutralization test

BEFV NAs were detected in the collected sera using the SN test [24]. The Israeli BEFV strain Yaqum-00 was used as the neutralized virus. Briefly, serum samples were diluted from 1:4 to 1:512 in a 0.05 ml/well volume using cell-growth medium in duplicates. Then, 0.05 ml/well of 100 TCID_50_/50 µl BEFV was added and the 96-well plates were incubated for 60 min at 37°C in a 5% CO_2_-humidified atmosphere. Vero cell suspension obtained from a monolayer culture by trypsinization was added at 3.5 × 10^5^ cell/ml in a 0.15-ml volume per well. DMEM supplemented with 10% heat-inactivated FBS and 1% antibiotic mix (penicillin, streptomycin, amphotericin B) was used as the growth medium. SN plates were maintained in an incubator at 37°C in a 5% CO_2_-humidified atmosphere and checked for the occurrence of cytopathic effects after 2 to 3 days. Each assay included a positive control serum with known NA titer as well as cow serum and fetal calf serum as negative controls. In each assay a virus control was titrated as well to ensure the use of 100 TCID50/50 µl. The same frozen virus stock was used for all assays. Antibody titers were expressed as the reciprocal of the highest initial serum dilution able to prevent cytopathic effect, starting with a 1:4 dilution. When cytopathic effect was prevented in only one of the two wells of the first dilution (1:4), the titer assigned to the sample was 1:2.

### 7: Statistical analysis

Chi-square test was used for calculating statistical significance of the comparison between rates of culling in vaccinated and non vaccinated groups. For each cow, we calculated the difference between rectal temperature on each of the 7 days after vaccination and the rectal temperature 3 days before vaccination. Milk production data were collected from the herd-management software records (NOA™, Israel Cattle Breeders Association). The average difference in the daily average milk production during the 6 days after vaccination and the 7 days before vaccination was calculated for each participating cow. The statistical significance of the differences in daily milk production and the rectal temperatures between the vaccinated and non vaccinated groups was calculated using an independent two-tailed t-test. The averages of the log of the NA titers in each vaccination category (i.e. volume of vaccine dose and number of vaccinations) on each blood-collection day were compared using either the independent two-tailed t-test or one-way ANOVA. To utilize all of the data available at different periods after vaccination, the NA titers of all sera collected after a certain number of vaccinations were compared, regardless of the final vaccination schedule administered to each particular cow. For example, the NA titers measured 1 month after the second vaccination in cows that ultimately received three vaccinations were included in the calculation of average titers 1 month after two vaccinations. For this comparison, the time gaps after last vaccination were grouped as follows: 0.5 month -13–15 days post last vaccination (PLV), 1 month—29 to 30 days PLV; 2 months—58 to 74 days PLV; 3 to 4 months—86 to 124 days PLV; 5 to 6 months—152 to 169 days PLV; 7 to 9 months—226 to 267 days PLV. Analysis was performed using SPSS 19.0 (Armonk, NY: IBM Corp.) In all comparisons. *P* < 0.05 was considered statistically significant. 

## Results

### 1: Safety

**Figure 2:**
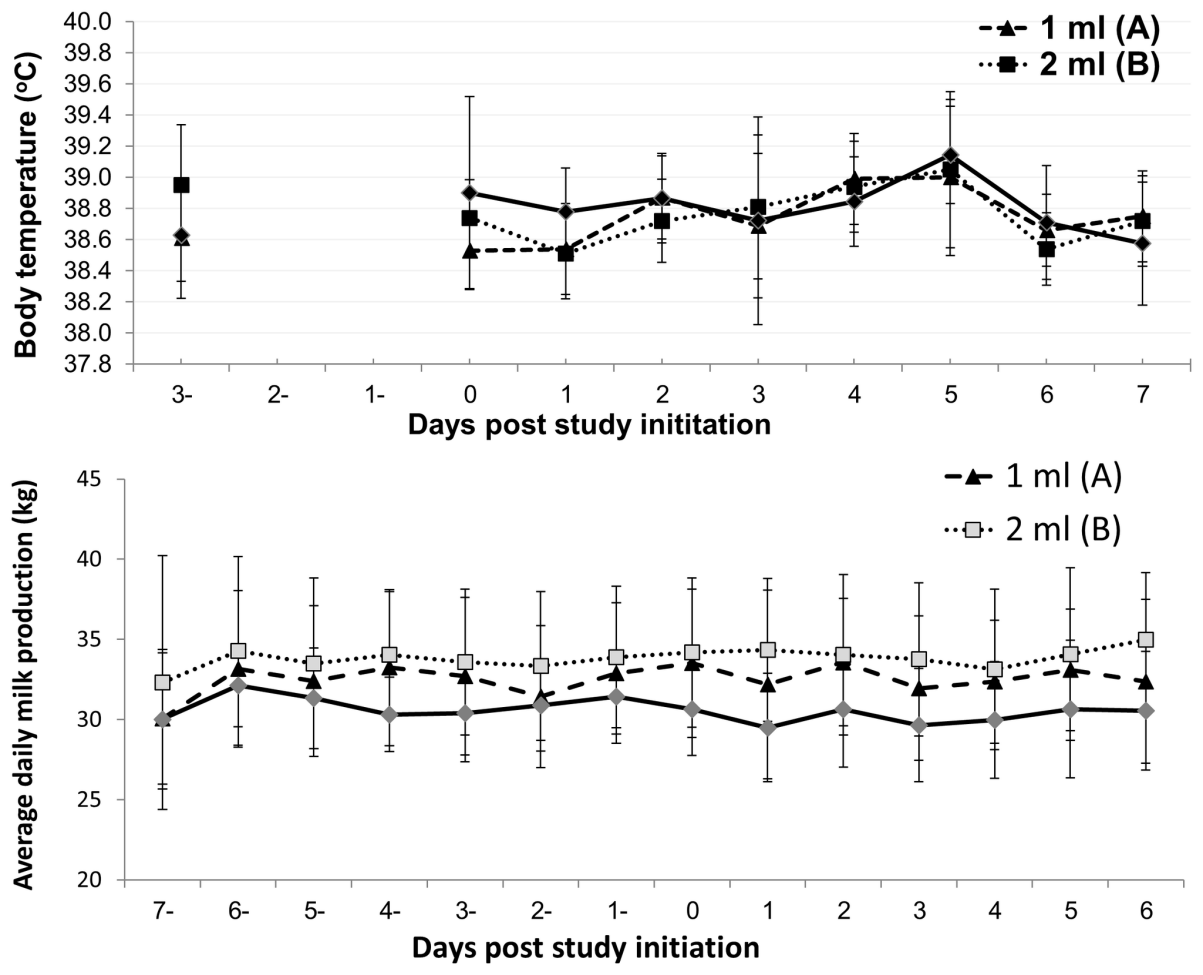
Daily measured rectal temperature (°C) and milk production (kg) pre vaccination and post vaccination. (I) Mean body temperature and (II) daily average milk production before and after vaccination with inactivated bovine ephemeral fever vaccine. Day 0: study initiation and first vaccination. Triangles: 1-ml vaccine. Squares: 2 -ml vaccine. Rhombuses: control group. Whiskers represent standard deviation for each group.

No adverse effects were observed after administration of the different vaccination protocols. Figure **2** summarizes the daily measured rectal temperature (°C) and milk production (kg) pre-vaccination and post-vaccination. No significant difference was observed between rectal temperatures measured in the vaccinated and control groups (Figure **2** **. I**). There were differences in the pre-vaccination average daily milk production between the vaccination groups. However, within each group, no significant change was observed in milk production after vaccination (Figure **2** **. II**). 

 Four, one and three cows were culled from the 1-ml dose, 2-ml dose and non vaccinated groups, respectively, between 102 days and 303 days PSI. Reasons for culling varied. None of them seemed to have any relation to vaccination. No statistically significant difference was documented in the rate of culling between the vaccinated and control cows (Table **1** **.**).

**Table 1:** List of cows culled from the herd during the study period.

Group	Cow number	Parity	Culling cause	Period between study initiation and culling (days)	No. of vaccine doses administered before culling	Period between last vaccination and culling (days)	*P*-value for the difference between groups
	1350	1	mastitis	163	1	163	
A (1 ml)	1330	1	abortion on day 185 of pregnancy	255	1	255	
	1346	1	infertility	255	1	255	
	1317	1	mastitis	303	1,2,3	36	*P*-value = 1
B (2 ml)	1338	1	mastitis	255	1	255	
	1318	1	traumatic reticulitis	102	NA	NA	
C (Control)	1348	1	infertility	255	NA	NA	
	1344	1	infertility	258	NA	NA	

*NA – not applicable

### 2: Effect of vaccine dose on NA titers

Two cows (6.7%) did not develop NA titers after the second vaccination. One of those cows was vaccinated twice, 9 months apart, with a 2-ml dose (group B1). The second non responding cow belonged to the group vaccinated in the second part of the study and receiving two doses, 1-ml each, one month apart (group D1). These cows were excluded from further analyses. 

Table **2** depicts the average neutralizing antibody (NA) fractional dilution after two injections, administered 1 month apart, of 1-ml or 2-ml of inactivated vaccine. The titers in the groups vaccinated twice at the beginning of the study and the group vaccinated twice in the second part of the study (9 months later) were compared as well (groups A1, B1 and D1, respectively). No significant difference was observed between antibody titers of the three groups 13-15 days or 29-30 days after administration of the second vaccine dose. Therefore, the cows that were vaccinated twice were combined into one group for further comparisons. 

**Table 2:** Comparison of average neutralizing antibody fractional dilution 13-15 and 29-30 days post 2nd vaccination with 1 ml or 2 ml vaccine dose.

	Protocol		Time elapsed from 2nd vaccination			
			13-15 days		29-30 days	
Dose	Research group	n	Average NA fractional dilution [CI95%]	*P*-value	Average NA fractional dilution [CI95%]	*P*-value
1 ml	A1	5	119 [47-300]	REF	45 [19-110]	REF
1 ml	D1	4	304 [136-683]	0.18	76 [24-243]	0.51
2 ml	B1	5	119 [32-446]	1	64 [28-147]	0.59

### 3: Duration of immunity

**Figure 3:**
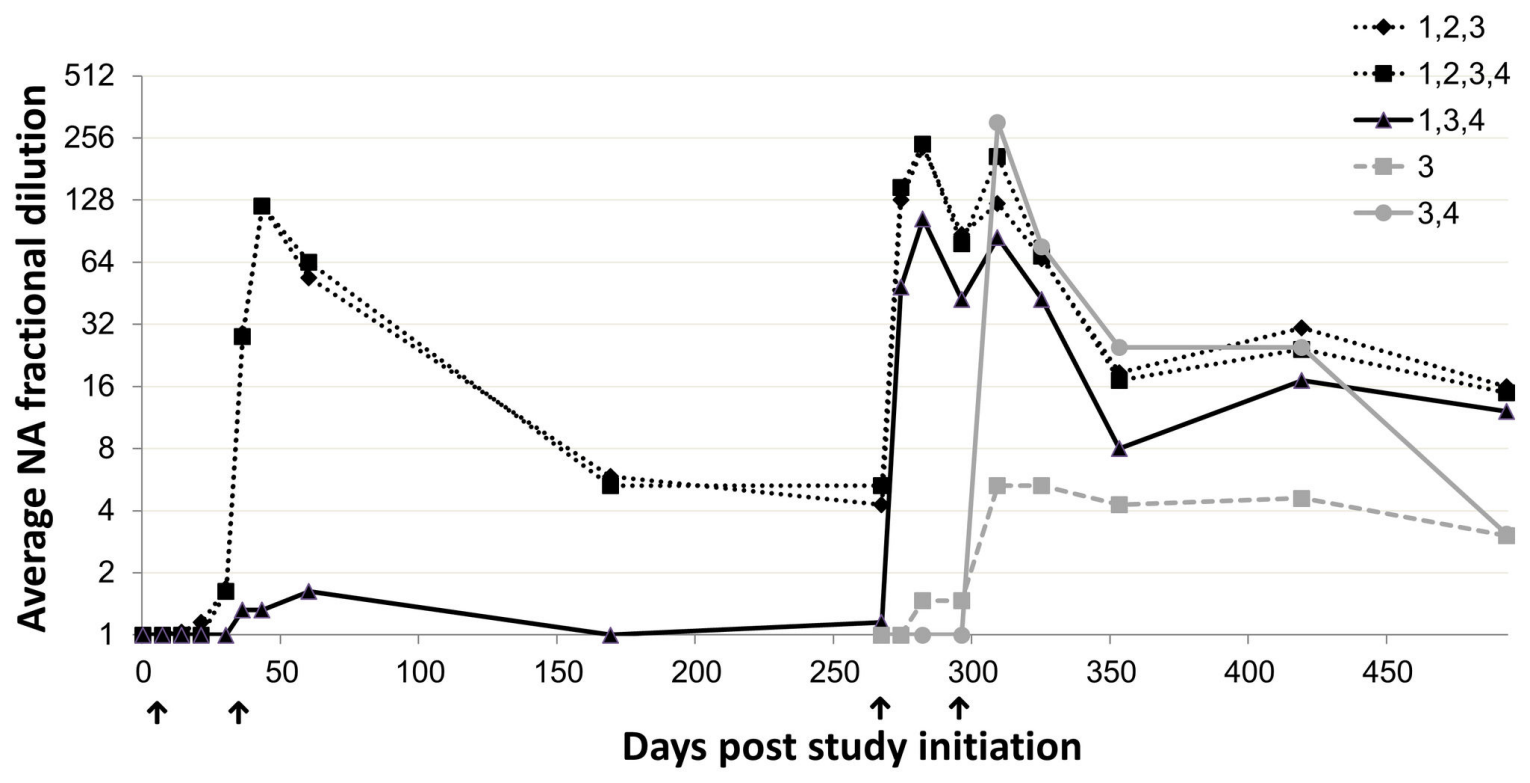
Average neutralization antibody (NA) fractional dilutions induced by vaccination with inactivated BEF vaccine according to five schedules. Arrows indicate vaccine administration on: 0, 30, 267 and 296 days, designated by numbers 1-4, respectively. Each vaccination schedule is designated by a unique line pattern or shape. The numbers near the line legend represent the vaccines administered to each group (e.g. 1,2,3 indicating vaccination on days 0,30 and 267 PSI).

A significant average rise in NA titer, reaching levels of 1:128 to 1:256, was observed 13-15 days after the second vaccination (1 ml or 2 ml). No titer difference was observed between cows vaccinated three or four times (Figure **3**.) Therefore, the cows originating from these two groups were combined into one group for further analysis and the NA titers measured in this combined group were compared with those elicited after two vaccinations. 

**Figure 4:**
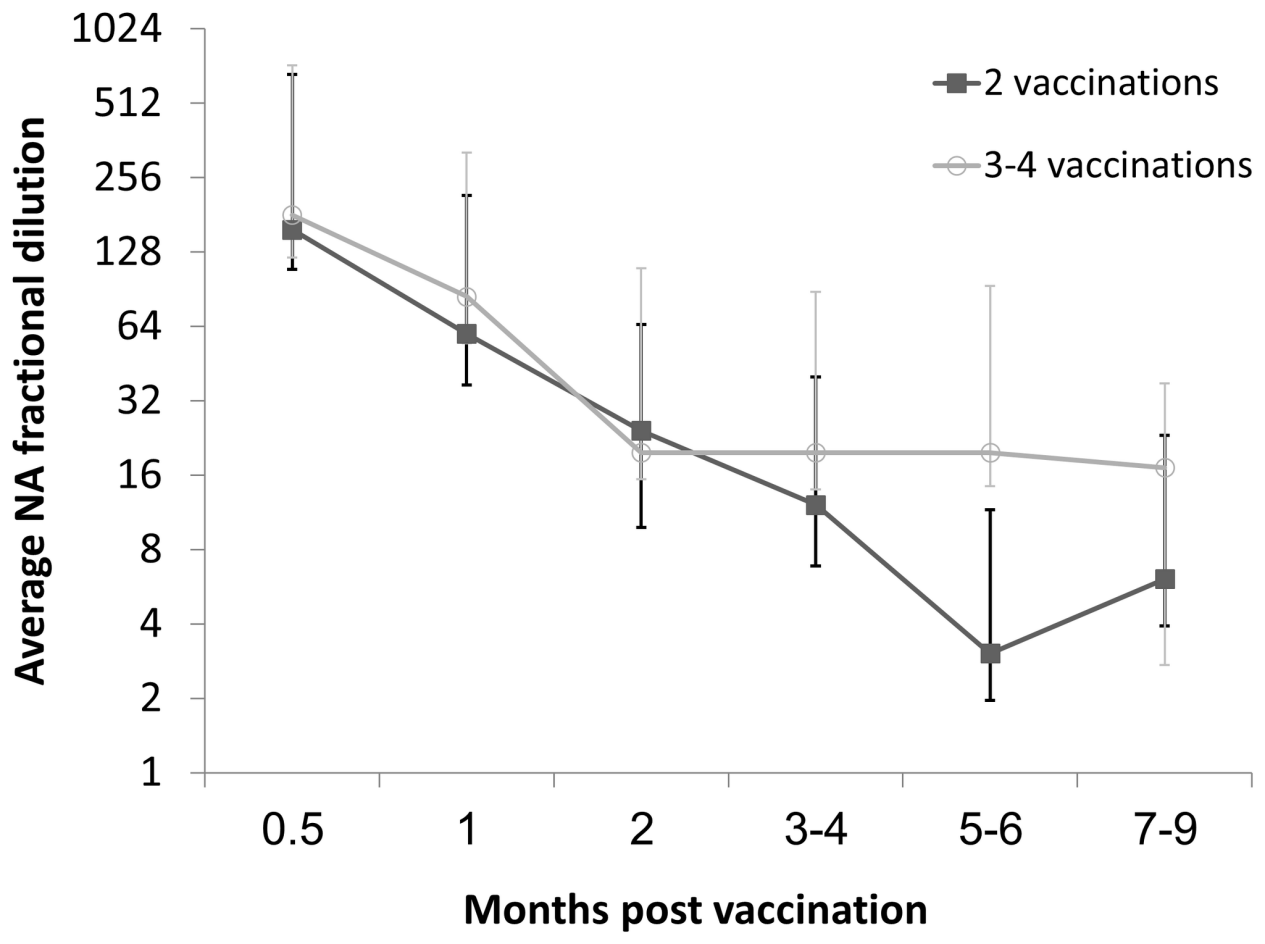
Average neutralization antibody (NA) fractional dilutions induced by two or three to four vaccinations with inactivated BEF vaccine. Black squares: two vaccinations, Grey circles: three to four vaccinations. Whiskers represent standard deviation for each group.

 No significant differences were found between the two groups in the NA titers measured up to 4 months PLV, though in both groups the average titer declined to approximately 1:16. The average NA titers measured 5–6 months after the last vaccination were significantly higher in the three- to four-vaccinations group than in the two-vaccinations group reaching 1:19.7 (CI_95%_=9.6-40.3) vs. 1:3 (CI_95%_=1.1-8.4), respectively. The same significant difference was demonstrated in the NA titers measured 7- 9 months after the last vaccination with an average NA titer of 17.1(CI_95%_=14.5-20.3) in the three- to four-vaccinations group compared to only 1:6.1 (CI_95%_=2.9-12.5) in the two-vaccinations group (Figure **4** **.**)

## Discussion

We tested the safety and immunogenicity an inactivated Israeli BEFV vaccine in cattle, and optimized a recommended protocol. The adjuvant used in this vaccine was water-in-oil-in-water (MONTANIDE ISA 206™)—a biphasic emulsion which is low in viscosity, easy to inject and does not induce granulomas at the injection site. In addition, this adjuvant has been found to stimulate longer immunity compared to aluminum salts adjuvant [16]. 

We investigated the NA response following several vaccine-administration protocols which varied in both the number and duration of the vaccinations. The vaccinated cows were followed for a period of 493 days. Until now, the longest consecutive follow-up period without challenge interference after inactivated BEFV vaccination was 6 months [8,16,25]. The study therefore provides insight into the long-term dynamics of the antibody response following vaccination with inactivated vaccines.

The results of this study indicate that the Israeli inactivated BEF vaccine is well tolerated and does not cause any adverse reactions when administered in either 1-ml or 2-ml doses. Culling rate did not differ between the various study groups. All culling events occurred a long time after vaccination. Reasons for culling did not seem to have any association with vaccination. Furthermore, no significant difference was observed between the rectal temperatures measured in the vaccinated and control groups. Post vaccination milk drop has been previously reported following the use of some killed vaccines [26], but to the best of our knowledge, it has never been studied for inactivated BEF vaccines. In this study we found no decrease in milk production as a consequence of the use of the inactivated vaccine. 

Antibody response was investigated by measuring NA titers specific to the virus used for vaccine preparation. Though exceptions may occur in some individual animals [15,27–29] high titers of specific antibodies elicited after vaccination are usually interpreted as correlated with protection from natural infection or challenge [30,31]. However, lack of standardization for the SN of BEFV complicates the attempt to directly infer protection from NA titers. In one study it was shown that titers higher than 1:45 are associated with protection from challenge [17]. The same authors later defined a titer of 1:64 as the upper limit of antibody titer associated with lack of protection [6] and others arbitrarily assigned a titer of 1:32 as a protective SN titer based on two serological surveys [25]. Yet it is not clear whether a correlation was found between this particular titer and protection. An adequate study, aimed at correlating NA titer with protection from challenge would have been helpful in interpreting the results presented in the current study.

NA levels of 1:128-1:256 were measured following two vaccinations with either 1- ml or 2- ml of vaccine. No significant difference in NA titers was measured after either three or four vaccinations. It can thus be concluded that there is no advantage in providing a fourth vaccination dose shortly after the third dose. The results of this study indicate that as in previous studies [8], an antibody decline to titers of 1:16-1:32 occurs 3–4 months after several vaccinations regardless of the number of vaccinations administered previously. However, while the NA titers measured 5–9 months after two vaccinations continued to decline, titers after three or four vaccinations remained stable and were significantly higher than in the two-vaccinations group. 

Overall, the administration of three or four doses of vaccine triggered a stable antibody response for a period of at least 7–9 months. A short duration of immunity after two vaccinations has been documented in other studies testing the immune response elicited by inactivated BEFV vaccines [8,25]. In this study, we show that administration of a third dose of inactivated vaccine can help overcome this problem by extending the duration of immunity. It is also worth noting that two cows (6.7% of the total number of vaccinated cows) did not respond to the vaccine. This information is likely to be of importance in further analyses of vaccine effectiveness.

In conclusion, we found the Israeli inactivated vaccine to be safe and immunogenic. The results of this study suggest that administration of two vaccinations of only 1-ml dose, one month apart and an additional single vaccination 9 months later may provide the longest immunological response. However, the titers measured at this period were significantly lower than the titer measured 1 month after vaccination. Thus, in order to provide immunity that will last the entire season it is recommended that the last vaccine will be administered a short time before the onset of BEF season. The results of this study should be complemented by results of an effectiveness study or by challenge data in order to formulate a solid recommendation protocol for use in cattle. 
